# NETosing Neutrophils Activate Complement Both on Their Own NETs and Bacteria *via* Alternative and Non-alternative Pathways

**DOI:** 10.3389/fimmu.2016.00137

**Published:** 2016-04-14

**Authors:** Joshua Yuen, Fred G. Pluthero, David N. Douda, Magdalena Riedl, Ahmed Cherry, Marina Ulanova, Walter H. A. Kahr, Nades Palaniyar, Christoph Licht

**Affiliations:** ^1^Cell Biology Program, The Hospital for Sick Children Research Institute, Toronto, ON, Canada; ^2^Program in Physiology and Experimental Medicine, The Hospital for Sick Children Research Institute, Toronto, ON, Canada; ^3^Department of Laboratory Medicine and Pathobiology, University of Toronto, Toronto, ON, Canada; ^4^Division of Haematology/Oncology, The Hospital for Sick Children, Toronto, ON, Canada; ^5^Division of Medical Sciences, Northern Ontario School of Medicine, Lakehead University, Thunder Bay, ON, Canada; ^6^Institute of Medical Sciences, University of Toronto, Toronto, ON, Canada; ^7^Department of Biochemistry, University of Toronto, Toronto, ON, Canada; ^8^Department of Paediatrics, University of Toronto, Toronto, ON, Canada; ^9^Division of Nephrology, The Hospital for Sick Children, Toronto, ON, Canada

**Keywords:** neutrophil extracellular traps, NETosis, complement system, alternative pathway, properdin, *Pseudomonas aeruginosa*

## Abstract

Neutrophils deposit antimicrobial proteins, such as myeloperoxidase and proteases on chromatin, which they release as neutrophil extracellular traps (NETs). Neutrophils also carry key components of the complement alternative pathway (AP) such as properdin or complement factor P (CFP), complement factor B (CFB), and C3. However, the contribution of these complement components and complement activation during NET formation in the presence and absence of bacteria is poorly understood. We studied complement activation on NETs and a Gram-negative opportunistic bacterial pathogen *Pseudomonas aeruginosa* (PA01, PAKwt, and PAKgfp). Here, we show that anaphylatoxin C5a, formyl-methionyl-leucyl-phenylalanine (fMLP) and phorbol myristate acetate (PMA), which activates NADPH oxidase, induce the release of CFP, CFB, and C3 from neutrophils. In response to PMA or *P. aeruginosa*, neutrophils secrete CFP, deposit it on NETs and bacteria, and induce the formation of terminal complement complexes (C5b–9). A blocking anti-CFP antibody inhibited AP-mediated but not non-AP-mediated complement activation on NETs and *P. aeruginosa*. Therefore, NET-mediated complement activation occurs *via* both AP- and non AP-based mechanisms, and AP-mediated complement activation during NETosis is dependent on CFP. These findings suggest that neutrophils could use their “AP tool kit” to readily activate complement on NETs and Gram-negative bacteria, such as *P. aeruginosa*, whereas additional components present in the serum help to fix non-AP-mediated complement both on NETs and bacteria. This unique mechanism may play important roles in host defense and help to explain specific roles of complement activation in NET-related diseases.

## Introduction

Neutrophils play a central role in the innate immune system and function in inflammation and immune surveillance. At sites of inflammation, neutrophils kill pathogens *via* phagocytosis and release of proteolytic enzymes (1, 2). Recently, the ability of neutrophils to form web-like neutrophil extracellular traps (NETs) has been identified as an additional strategy for antimicrobial defense. The process of NET formation (i.e., NETosis) is a specific form of cell death, in which nuclear DNA undergoes decondensation with subsequent expulsion of chromatin that is coated with cytotoxic granular proteins, such as myeloperoxidase (MPO), elastase, and other proteases (3). NETs are released in response to a variety of stimuli, including NADPH oxidase (Nox) agonist, such as phorbol-12-myristate-13-acetate (PMA), inflammatory stimuli, and bacteria (4, 5). Two major types of NETosis have been reported to date: Nox-dependent NETosis and Nox-independent NETosis, in which reactive oxygen species (ROS) are generated by Nox and mitochondrial complexes, respectively (6–9). In both of these types of NETosis, neutrophil release chromatin coated with granular proteins as NETs. In the presence of C5a, GM-CSF-primed neutrophils undergo a vital NETosis, in which cells do not die, but release mitochondrial DNA. This type of NETosis is regulated by mitochondrial ROS production (10).

Once formed, NETs ensnare pathogens and expose them to high localized concentrations of antimicrobial proteins (11). NETs can also be cytotoxic and have been shown to contribute to thrombosis, sepsis, cystic fibrosis, asthma, systemic lupus erythematosus (SLE), rheumatoid arthritis (RA), and anti-neutrophil cytoplasmic antibody (ANCA)-associated vasculitis (AAV) (12–24). Complement and infections have been implicated in the pathogenesis and exacerbation of many of these diseases. Although it has recently been described that NETs can activate and deposit complement alternative pathway (AP) components (25), the involvement of the different complement pathways and their components in the context of NETosis and bacterial infection has not been fully understood. This fundamental knowledge is essential for understanding molecular mechanisms involved in NET-related pathobiology.

The complement system consists of more than 30 proteins distributed in the circulation and on endothelial cells, and functions primarily in microbial defense and clearance of immune complexes and injured cells (26). Complement can be constantly active (*via* the complement AP) or become activated by immune complexes and dying cells [*via* the C1q-mediated classical pathway (CP)] or carbohydrate ligands on microorganisms [*via* the lectin pathway (LP)] (26). Complement factor P (CFP), the only positive complement regulator, acts as stabilizer of the AP convertase (C3bBbP) and selective pattern recognition molecule of certain microorganisms and host cells (i.e., apoptotic/necrotic cells) by serving as a platform for the assembly of the AP C3 convertase (27). Complement progression includes the activation of complement proteins C3 and C5 (to form the potent anaphylatoxins C3a and C5a and the opsonins C3b and C5b) and the subsequent activation of the terminal pathway with the formation of the potentially lytic membrane attack complex (MAC), C5b–9. AP activation is critically enhanced by the C3 convertase C3bBbP, and requires tight regulation to maintain the balance between necessary activation and harmful over-activation (26). Bacteria are capable of inducing Nox-dependent NETosis (9, 28–30), and we aimed to identify possible links between NETosis, bacteria, and the complement system, in particular, the possibility that neutrophils mount a targeted complement response to infectious agents *via* the formation of NETs and deposition of complement components on NETs and microbial pathogens.

## Materials and Methods

### Ethics

Informed written consent was obtained from all donors. The study protocol was approved by the Research Ethics Board at The Hospital for Sick Children, Toronto, ON, Canada.

### Reagents

All buffer salts and reagents were obtained from Sigma-Aldrich (St. Louis, MO, USA) unless stated otherwise.

### Bacterial Culture

*Pseudomonas aeruginosa* (mPA01, PAKwt, and PAKgfp) cultures were grown overnight in LB-broth. PAKgfp was maintained in 30 μg/ml gentamicin. The concentration of bacteria was calculated using [CFU] × 10^8^ = (OD_600_) 30.88 − 99,607. For NETosis assays, *P. aeruginosa* sub-cultured for 3 h was used at a multiplicity of infection (MOI) of 10 or 100.

### Neutrophil Isolation and Preparation of Neutrophil Lysates

Human peripheral neutrophils were purified from whole blood (20 ml) collected in BD EDTA-vacutainers from healthy donors using Polymorphprep™ (Axis-shield, Oslo, Norway). After lysing erythrocytes with hypotonic buffer, neutrophils were resuspended in RPMI 1640 (Wisent Bioproducts, Montreal, QC, Canada) (31). To obtain neutrophil lysate, cell pellets were resuspended in a lysis buffer [1% (v/v) Triton X-100, 50 mM Tris, pH 7.4, 10 mM KCl containing 2× complete, mini protease inhibitor cocktail (Roche Diagnostics, Laval, QC, Canada) supplemented with 0.5 mM EDTA, 25 μM leupeptin, 25 μM pepstatin, 25 μM aprotinin, 1 mM levamisole, 1 mM Na_3_VO_4_, 25 mM NaF, 1 mM PMSF], sonicated (VWR Sonics model 50D), and incubated for 15 min at 4°C. Neutrophil lysates were centrifuged at 25,000× *g* for 30 min at 4°C and stored at −80°C for future analysis.

### Neutrophil Activation, Oxidative Burst, and Secretion

Neutrophils (2 × 10^7^ cells/ml) were resuspended in RPMI 1640 with 10 mM Hepes, pH 7.4, and activated with C5a (CompTech, Tyler, TX, USA) (1 μM), formyl-methionyl-leucyl-phenylalanine (fMLP) (1 μM) or PMA (20 nM), and incubated [37°C, 5% (v/v) CO_2_] for 30 min. Stimulation was terminated by incubating these cells at 4°C for 5 min. Neutrophils were pelleted (1000× *g* for 10 min) and the supernatant was collected and further centrifuged at 25,000× *g* for 10 min at 4°C, immediately placed in 2× neutrophil Laemmli sample buffer, heated at 95°C for 5 min and stored at −80°C for future analysis. Neutrophil lysates were prepared from the remaining cell pellet as described in the Section “Neutrophil Isolation and Preparation of Neutrophil Lysates.” To determine respiratory burst, neutrophils (1 × 10^6^ cells/ml) were pre-loaded with dihydrorhodamine (DHR) 123 (10 μM), treated with the agonists as above and analyzed by flow cytometry (Gallios, Beckman Coulter, Mississauga, ON, Canada). Cells were first gated with forward and side scatters, and further gated for Hoechst (1 μg/ml) using 405/450 BP 40 filter channel. ROS was detected using 488/429 BP 28.25 filter channel.

### Sytox Green Plate Reader Assay

Neutrophils (3 × 10^4^ cells) were seeded onto 96-well plates in the presence of cell-impermeable Sytox Green DNA-binding dye (5 μM) and were activated with agonists or three stains of *P. aeruginosa* (mPAO1, PAKwt, and PAKgfp) at MOIs of 10 and 100. For inhibition studies, neutrophils were preincubated with 2 μM of Nox inhibitor, diphenyleneiodonium (DPI) for 1 h before activation. Fluorescence intensity was measured by the POLARstar Omega microplate reader (BMG Labtech, Ortenberg, Germany) with excitation/emission (485/520), every 30 min. NET formation was normalized to total neutrophils DNA content determined by permeabilizing the cells with 0.5% (w/v) Triton X-100.

### Western Blot

Neutrophil lysates (50 μg) were size-fractionated in 10% (w/v) SDS-polyacrylamide gels, transferred to nitrocellulose membranes, blocked with 5% (w/v) skim-milk + 0.05% (v/v) Tween-20 (TBST), probed with goat polyclonal antibody to complement proteins (1:1000 dilution; Complement Technology, Tyler, TX, USA) or mouse monoclonal antibody to β-actin (BA3R, 1:10,000 dilution; Thermo Fisher Scientific, Rockford, IL, USA) in 5% (w/v) skim-milk in TBST, washed, and incubated with secondary antibody in 5% (w/v) skim-milk in TBST. Proteins were detected using Western Lighting™ Plus-ECL, Enhanced (PerkinElmer, Waltham, MA, USA) and developed on radiographic film on a Kodak X-Omat 2000a processor.

### Detection of Complement Proteins in NETs

Phorbol-12-myristate-13-acetate (20 nM) activated neutrophils at 240 min were fixed with 4% (w/v) paraformaldehyde (Electron Microscopy Sciences, Fort Washington, PA, USA), blocked with 3% (v/v) cold water fish skin gelatin and incubated with anti-complement antibodies: rabbit polyclonal antibody to CFP (SC-68366, 1:50 dilution; Santa Cruz Biotechnology, Dallas, TX, USA), rabbit polyclonal antibody to complement factor B (CFB) (SC-67151, 1:50 dilution; Santa Cruz Biotechnology, Dallas, TX, USA), and rabbit polyclonal antibody to complement C3 (ab97462, 1:100 dilution; Abcam, Cambridge, MA, USA). Neutrophils activated with PMA were co-stained with mouse monoclonal antibody to MPO (ab25989, 1:500 dilution; Abcam, Cambridge, MA, USA). Complexes were detected with donkey anti-primary antibodies conjugated with Alexa Fluor^®^ 555 (Invitrogen, Eugene, OR, USA). Specimens were mounted with Dako Fluorescence Mounting Media (Dako Canada, Burlington, ON, Canada) for analysis with spinning-disk confocal microscopy.

### C5b–9 Formation on NETs

After 240 min of neutrophil activation, culture plates were centrifuged at 200× *g* for 5 min at 4°C. The media was replaced with 500 μl of 20% (v/v) Refludan^®^ (Bayer Healthcare, Wayne, NJ, USA) fresh frozen PPP prepared in RPMI 1640 media or AP buffer (20 mM Hepes, pH 7.4, 144 mM NaCl, 7 mM MgCl_2_, and 10 mM EGTA). For experiments with AP buffer, a buffer exchange was performed prior to the addition of plasma (Bayer Healthcare, Wayne, NJ, USA). After 15 min [37°C, 5% (v/v) CO_2_], specimens were fixed with 4% (w/v) paraformaldehyde (Electron Microscopy Sciences, Fort Washington, PA, USA). In some experiments, DNase I (50 μg/ml) was added to digest DNA. After blocking with 3% (v/v) gelatin, mouse monoclonal antibody to C5b–9 (DIA 011-01, 1:200 dilution; Antibody Shop, Gentofte, Denmark) was incubated, washed, and further incubated with donkey anti-mouse secondary antibody conjugated with Alexa Fluor^®^ 555 (Invitrogen, Eugene, OR, USA). PAKgfp signal was enhanced with Alexa Fluor^®^ 488 conjugated rabbit polyclonal antibody to GFP (A21311, 1:400 dilution; Invitrogen, Eugene, OR, USA). For CFP inhibitor assays, optimal concentration of a mouse monoclonal anti-CFP antibody (Anti Factor P#1, A233; Quidel Corporation, San Diego, CA, USA) was determined by rabbit red blood cell lysis assay (32). An antibody concentration of 4 μg/ml was used in the final assays. Presence of C5b–9 was detected as described above. Wheat germ agglutinin was used for labeling neutrophil membrane. Specimens were mounted and analyzed with spinning-disk confocal microscopy.

### Spinning-Disk Confocal Microscopy and Colocalization Analysis

Images were taken on an Olympus IX81 inverted fluorescence microscope using a 60×/1.35 oil immersion objective equipped with a Hamamatsu C9100-13 back-thinned EM-CCD camera and Yokogawa CSU X1 spinning-disk confocal scan head (with Spectral Aurora Borealis upgrade). The unit is equipped with four separate diode-pumped solid state laser lines (Spectral Applied Research, 405, 491, 561, and 642 nm) with emission filters: 447 ± 60, 525 ± 50, 593 ± 40, 620 ± 60, 676 ± 29, and 700 nm ± 75, and 1.5× magnification lens (Spectral Applied Research). Confocal images were taken with an Improvision Piezo Focus Drive. *Z*-stacks were taken at 0.25 μm. Images taken using the spinning-disk confocal microscope were deconvolved by iterative restoration using Volocity Software (PerkinElmer, Waltham, MA, USA) with confidence limit set to 95% and iteration limit set to 20.

### Statistical Analysis

Student’s *t*-test, or one-way or two-way ANOVA with Tukey’s multiple comparison test was used for statistical comparison as needed. A *p*-value was set at 0.05, 0.01, or 0.001 for statistical significance. All statistical analyses were performed using GraphPad Prism (GraphPad Software, La Jolla, CA, USA) statistical analysis software (Version 6.0).

## Results

### PMA, but not fMLP and C5a, Induces NETosis

Reactive oxygen species is considered to be important for NETosis. However, different agonists induce ROS to different degrees. Therefore, to determine whether ROS was sufficient to induce NETosis, ROS production was measured 30 min post neutrophil stimulation using DHR 123 and flow cytometry. C5a (1–2 μM) did not generate ROS above baseline levels (Figure S1A in Supplementary Material); however, similar concentrations of fMLP led to a 1.5-fold increase in ROS compared to baseline values (*p* < 0.05; Figure S1B in Supplementary Material). The use of 20 nM PMA produced a ninefold increase of ROS in comparison to non-treated control neutrophils (*p* < 0.05; Figure S1C in Supplementary Material). The overall ability of these agonists to induce ROS production was PMA >> fMLP > C5a.

**Figure 1:**
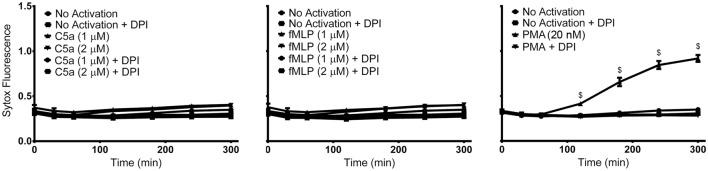
**PMA, but not C5a and fMLP, induce NET formation**. Sytox Green fluorescence plate reader assay for neutrophils activated with C5a (1 or 2 μM), fMLP (1 or 2 μM), and PMA (20 nM) reveals NET formation only after stimulation with PMA. Diphenyleneiodonium (DPI), a NADPH oxidase inhibitor, blocks PMA-induced NET formation. Data are presented as mean ± SEM from three to seven individual experiments. Fluorescence emission of Sytox Green was normalized to total DNA of resting neutrophils permeabilized with 0.5% (v/v) triton X-100. Statistical significance was obtained by comparing to SYTOX fluorescence from resting neutrophils. Two-way ANOVA with Tukey’s multiple comparison test, ^$^*p* < 0.0001.

Although both PMA and fMLP induced ROS production, whether fMLP can induce NETosis is uncertain. Therefore, to identify the ability of PMA, fMLP, and C5a to independently elicit NETosis, we treated neutrophils with varying concentrations of these reagents. NETosis was monitored using a plate reader assay by measuring the production of extracellular DNA. This assay monitors the fluorescence generated by the binding of cell-impermeable DNA-binding Sytox Green fluorescent dye to NET DNA. Neither C5a nor fMLP (up to concentration of 2 μM) induced NET formation within the observed 300-min time period (Figure 1). However, stimulation of neutrophils with PMA resulted in NET generation after approximately 120 min, as determined by Sytox Green plate reader assay (Figure 1) and nuclear morphology changes (Figure S2 in Supplementary Material). As expected, the use of the NADPH inhibitor DPI abrogated PMA-induced NET formation with levels remaining near baseline. This confirms that PMA induces NETosis *via* the Nox-dependent pathway, and that C5a and fMLP on their own do not induce NETosis.

### Induction of NETosis Causes Release of Complement Factors

**Figure 2:**
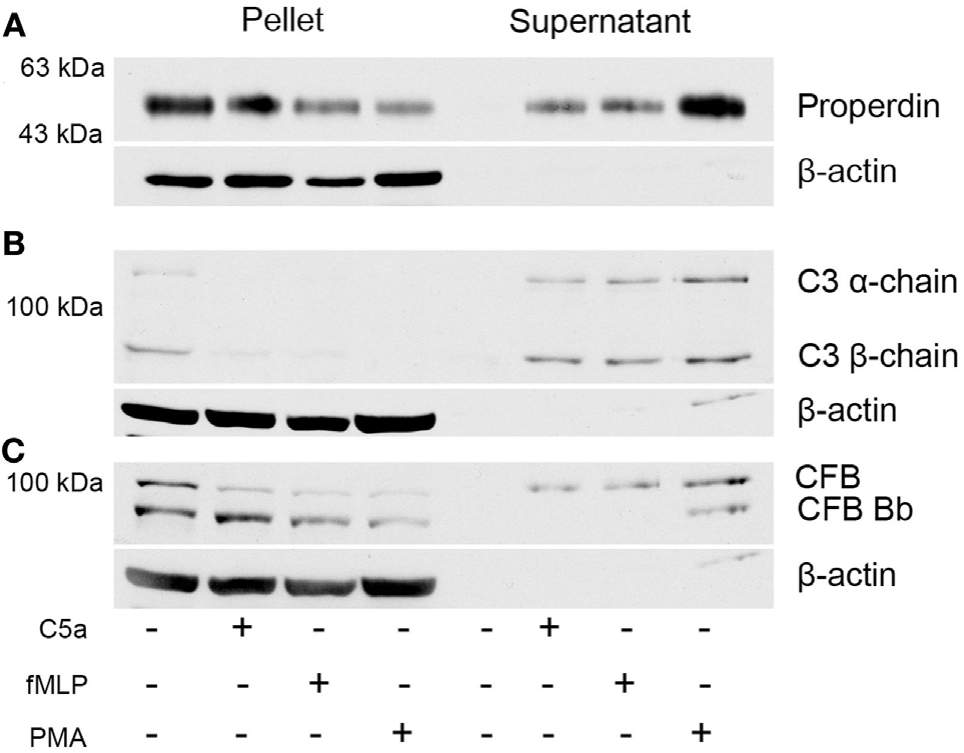
**Neutrophils secrete complement proteins upon activation with PMA, fMLP, and C5a**. Neutrophils were activated with C5a (1 μM), fMLP (1 μM), and PMA (20 nM) for 30 min. Cells were harvested by centrifugation, and the supernatant was collected to investigate for secreted proteins. Western blot analysis of the pellet and the supernatant reveals that neutrophils contain **(A)** CFP, **(B)** C3, and **(C)** complement factor B and secrete them upon activation. β-actin served as loading control.

To identify whether stimulation of neutrophils results in the release of complement factors, Western blot analysis was performed on the cell pellets and cell-free supernatant (Figure 2). Protein levels of the complement proteins CFP, C3, and CFB were compared for neutrophils induced for 30 min either with C5a, fMLP, and PMA or with buffer. Complement proteins were not detected in the supernatant of non-activated neutrophils, with proteins being identified exclusively in cell pellet samples. The release of all three complement proteins was observed for all induction methods; however, the use of PMA elicited the greatest release of both CFP and CFB within the supernatant (Figures 2A–C). Pelleted samples for activated cells also contained a large amount of complement proteins: primarily CFP and CFB (and its activation product Bb). Furthermore, CFB-Bb was only identified in PMA-induced neutrophils (Figure 2C). These results indicate the capability of PMA to not only induce NETosis but also cause the greatest release of complement proteins in comparison to C5a and fMLP.

### PMA-Induced NETosis Deposits CFP on NETs

**Figure 3:**
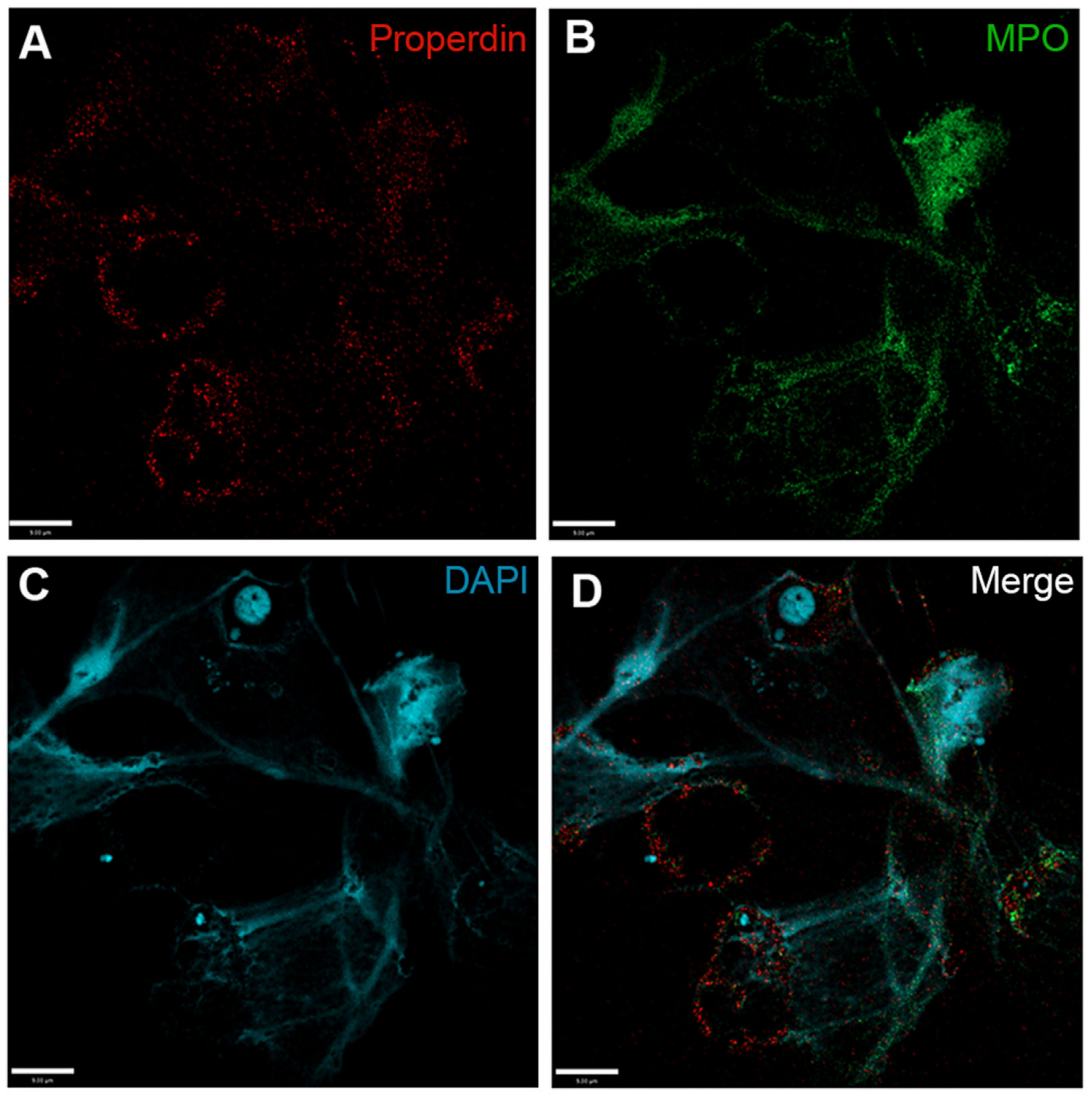
**Complement factor P deposits on PMA-induced NETs**. Spinning-disk immunofluorescence microscopy images show that **(A)** complement proteins, e.g., CFP (red), are found to associate with cell body, and NETs induced by PMA. NETs were visualized using **(B)** an anti-MPO antibody (green) and **(C)** DAPI stain for DNA (blue). **(D)** Merged image shows relative locations of proteins on cell body and NETs. Images were taken using a 60×/1.35 oil immersion objective. Scale bar, 9.00 μm.

As the next step, we sought to identify whether CFP was capable of adhering to NETosing neutrophils and NETs. Immunofluorescence analysis performed on PMA-induced NETs showed the deposition of both CFP and MPO (another known NET-associated protein) on the surface of extracellular NETs (Figure 3). CFP could also be detected on the neutrophil membranes after NET formation. Deposition pattern of both MPO and CFP shows substantial overlap throughout the extracellular DNA lattice structure. Therefore, neutrophils deposit CFP on NETs.

### PMA-Induced NETs Activate Complement

**Figure 4:**
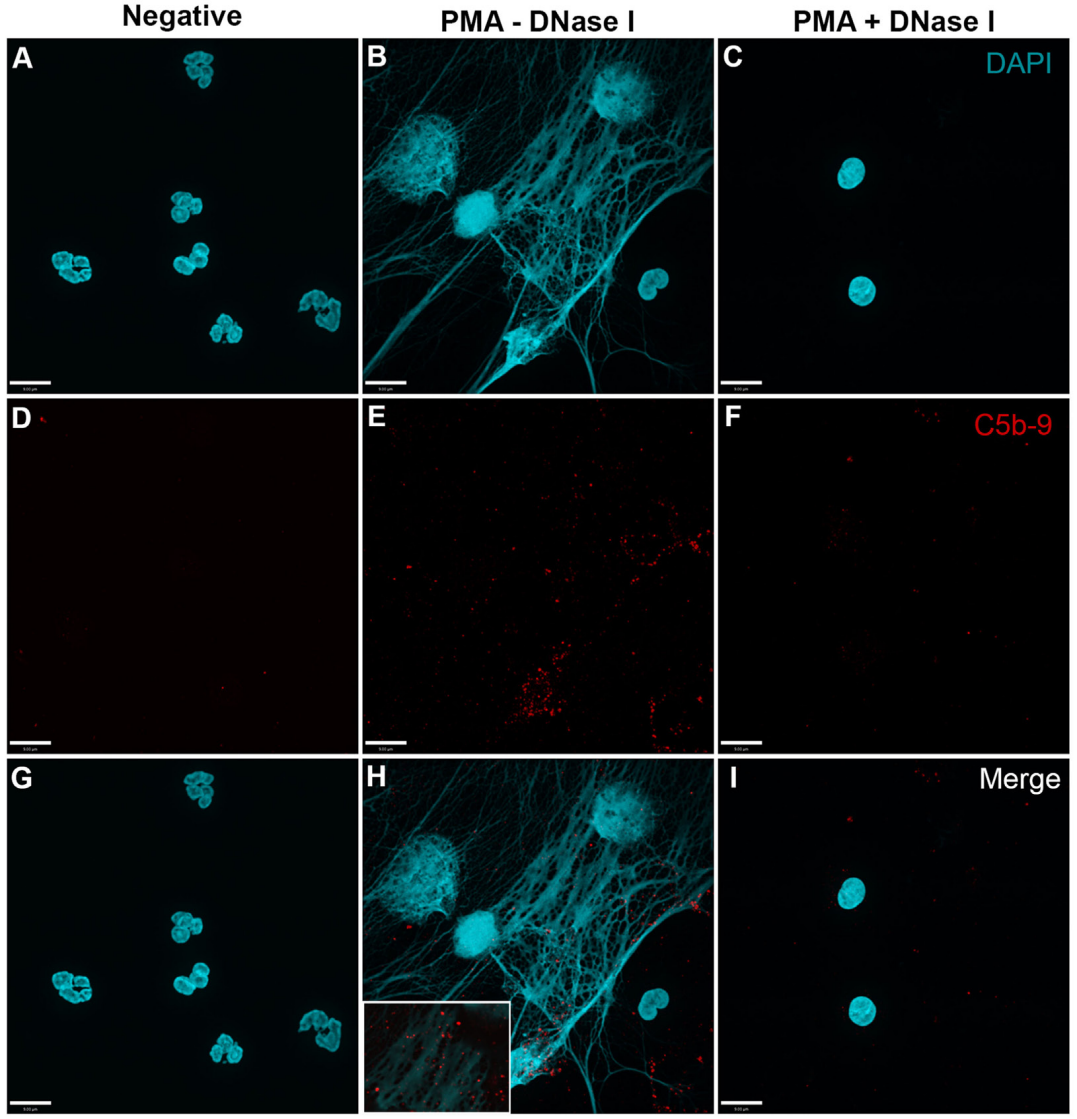
**PMA-induced NETs activate complement to form C5b–9**. Neutrophils were left untreated **(A,D,G)**, activated with 20 nM PMA **(B,E,H)**, and treated with DNase I after PMA activation to disseminate the formation of NETs **(C,F,I)**. After induction of NETosis in RPMI buffer, 20% (v/v) autologous plasma was added. **(A–C)** NETs were visualized with DAPI. **(D–F)** C5b–9 was detected using mouse monoclonal antibody to C5b–9. Image was taken with 60×/1.35 oil immersion objective. **(H)** with 2× magnified inset of representative area. Scale bar, 9.00 μm.

**Figure 5:**
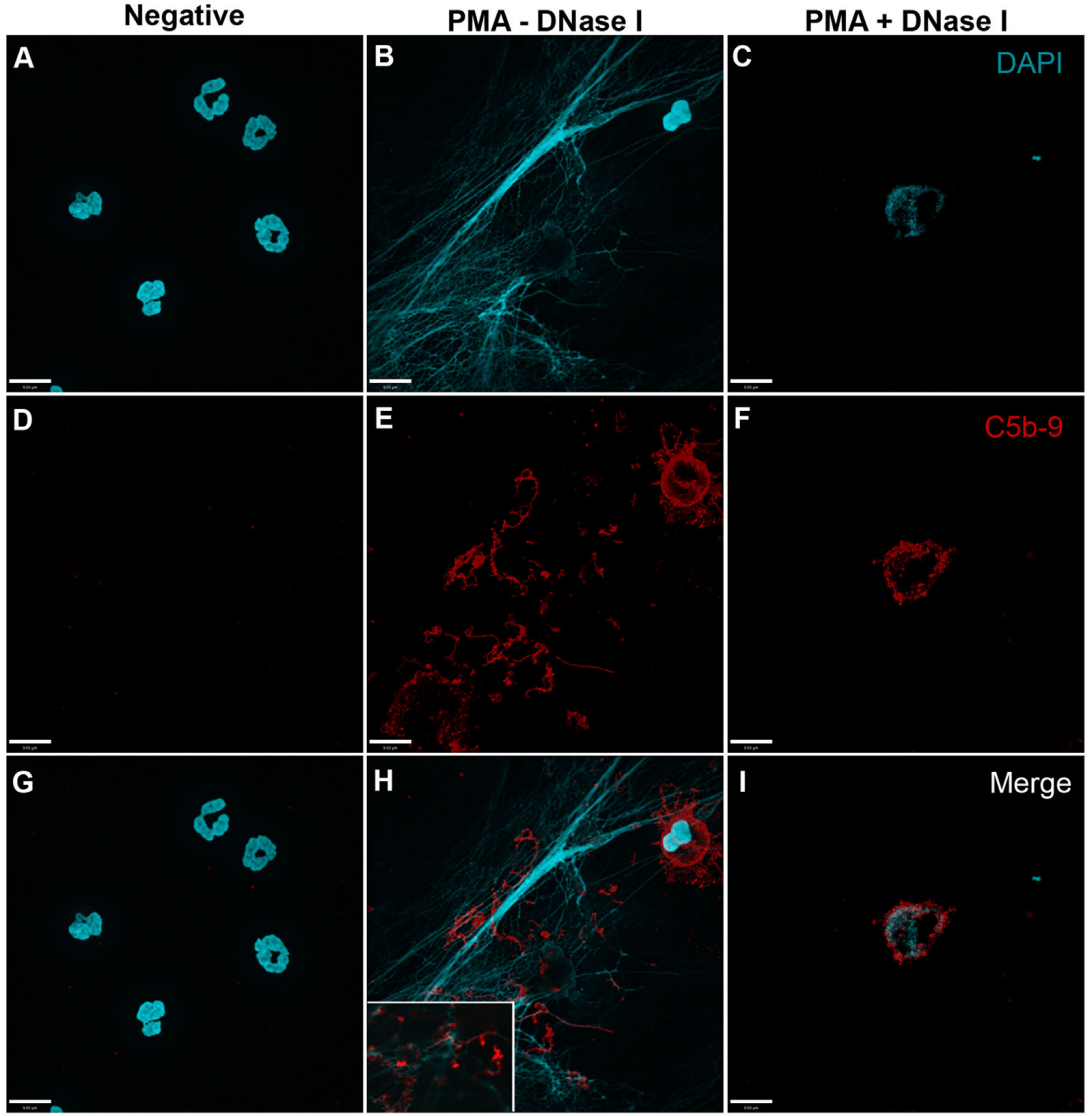
**PMA-induced NETs activate complement alternative pathway to form C5b–9**. Neutrophils were left untreated **(A,D,G)**, activated with 20 nM PMA **(B,E,H)**, and treated with DNase I to disseminate the formation of NETs **(C,F,I)**. A buffer exchange was performed with three washes of PBS and one wash of AP buffer, followed by addition of 20% (v/v) autologous plasma in AP buffer. **(A–C)** NETs were visualized with DAPI. **(D–F)** C5b–9 was detected using mouse monoclonal antibody to C5b–9. Image was taken with 60×/1.35 oil immersion objective. **(H)** with 2× magnified inset of representative area. Scale bar, 9.00 μm.

In order to analyze whether complement activation occurs on NETs, neutrophils were stimulated with PMA, washed and incubated with 20% complement active plasma for 15 min in complement competent buffers. Under these conditions, both AP- and non-AP-mediated complement fixation can occur. Immunofluorescence microscopy was performed on these NETs to identify the deposition of terminal complement complex C5b–9. Images show that C5b–9 deposits on NETs (Figures 4 and 5 – middle column; Figures 4H and 5H with 2× magnified insets of representative areas). The use of DNAse I, which removes pre-formed NETs, causes a large decrease in the detection of C5b–9 deposition. These data suggest that the presence of NETs is necessary to activate complement cascade and deposit terminal complement complex.

### PMA-Induced NETosis Activates Complement Also *via* Alternative Pathway

**Figure 6:**
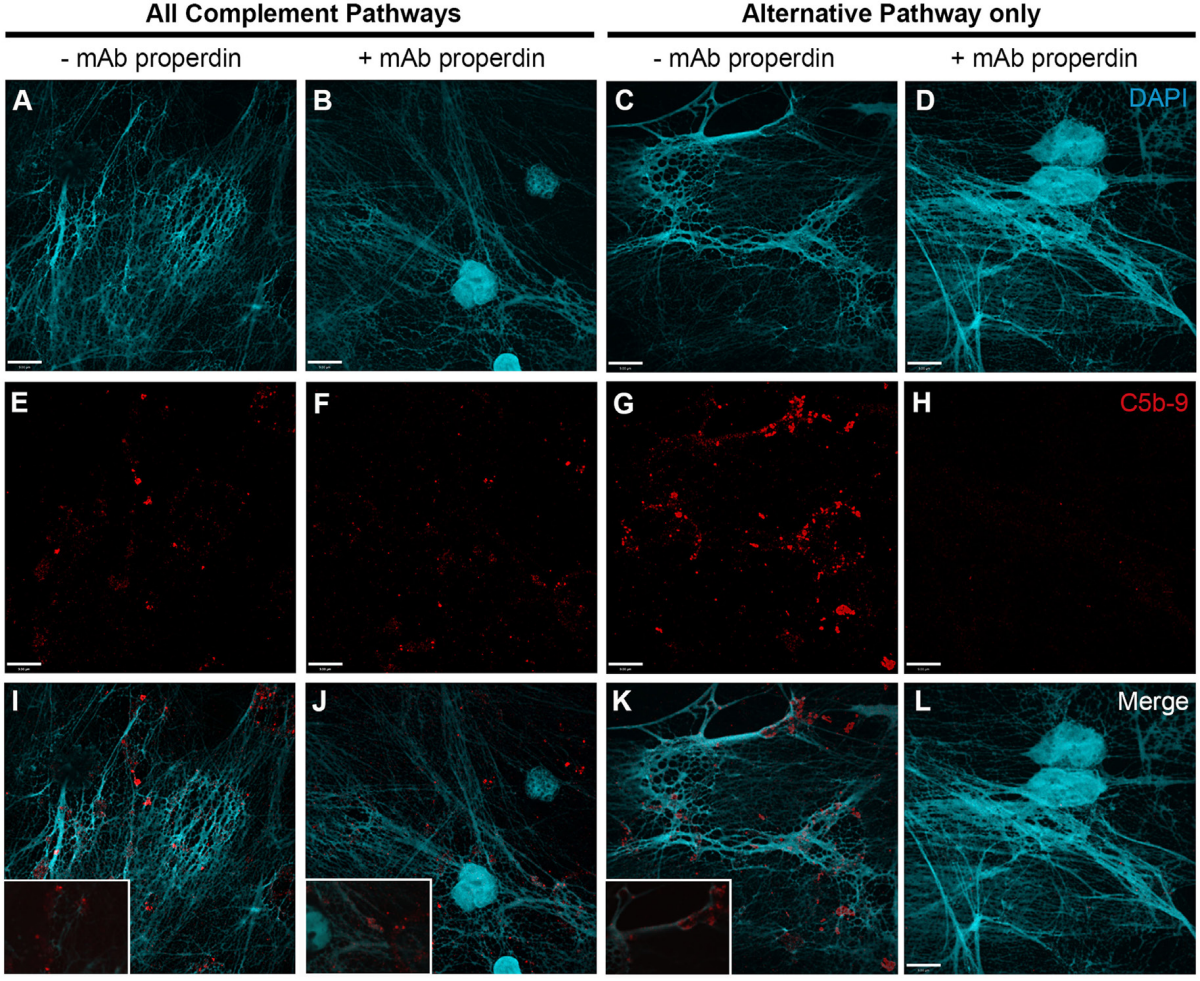
**Anti-CFP antibody blocks activation of complement AP to form C5b–9 on PMA-induced NETs**. Neutrophils were activated with PMA (20 nM) to induce NET formation. Plasma with RPMI buffer was used to activate all complement pathways, AP buffer to only activate the alternative pathway. A monoclonal anti-properdin antibody was used to inhibit AP activation. A buffer exchange was performed followed by addition of 20% (v/v) plasma:RPMI1640 + 10 mM Hepes [far left column **(A,E,I)**], 20% (v/v) plasma:RPMI1640 + 10 mM Hepes + mAb properdin [left column **(B,F,J)**], 20% (v/v) plasma:AP buffer [right column **(C,G,K)**], 20% (v/v) plasma:AP buffer + mAb properdin [far right column **(D,H,L)**]. **(A–D)** show DAPI, **(E–H)** C5b–9 deposition, and **(I–L)** merged images. Images were taken with 60×/1.35 oil immersion objective. **(I–K)** with 2× magnified insets of representative areas. Scale bar, 9.00 μm.

To further identify the contribution of AP to NETosis, this experiment was repeated except that the plasma incubation step was performed in the presence of AP buffer allowing for AP activation only. Immunofluorescence microscopy images show that C5b–9 could be deposited *via* AP (Figure 6; Figures 6I–K with 2× magnified insets of representative areas). To determine whether blocking CFP is sufficient to prevent complement activation, 4 μg/ml anti-CFP antibody was added during complement activation. This antibody concentration was chosen because AP-dependent rabbit erythrocyte hemolysis was inhibited in the presence of >4 μg/ml anti-CFP antibody (Figure S3 in Supplementary Material). Immunofluorescence microscopy reveals that blocking CFP fully prevents C5b–9 deposition in AP buffer conditions, but reduced C5b–9 deposition only slightly in complete buffer conditions (Figure 6). Therefore, both AP- and non-AP-mediated complement depositions occur on NETs induced by PMA.

### *P. aeruginosa*-Induced NETosis Deposits CFP on NETs and Bacteria

**Figure 7:**
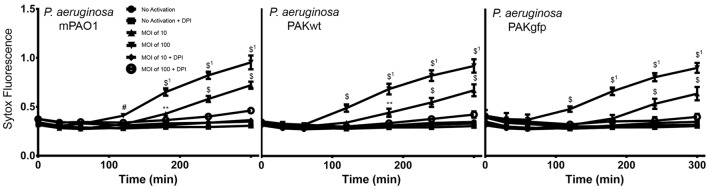
***P. aeruginosa* induces Nox-dependent NETosis**. Sytox Green plate reader assay showing the NETosis ability of different strains of *P. aeruginosa* (mPAO1, PAKwt, and PAKgfp). Fluorescence emission of Sytox green was normalized to total DNA of resting neutrophils permeabilized with 0.5% triton X-100. Nox inhibitor DPI suppresses the NETosis of all three strains. Data are presented as mean ± SEM from four to seven individual experiments. Statistical significance compared to Sytox fluorescence from resting neutrophils. ^1^ denotes statistical significance between different multiplicities of infection. Two-way ANOVA with Tukey’s multiple comparison test, *p < 0.05, **p < 0.01, ^#^*p* < 0.001, ^$^*p* < 0.0001.

To test the ability of complement fixation on pathogens during NETosis, neutrophils were exposed to various strains of *P. aeruginosa* (mPA01, PAKwt, and PAKgfp). Similar to the PMA experiments described above, NETosis was monitored using Sytox Green plate reader assays. All three strains of these bacteria induced NETosis, and followed similar kinetics in terms of post-infection time response (Figure 7). NETosis began at approximately 120 min and continued to increase throughout the 300-min experimental time period. Furthermore, this induction is bacterial load dependent with both a faster response and a larger response observed when the MOI was increased from 10 to 100. To determine whether this NETosis induction was dependent on Nox, DPI was included in the media. Nox inhibitor DPI fully abrogated *P. aeruginosa*-induced NETosis for all three stains at both MOIs (*p* < 0.05). This finding indicates that similar to PMA, bacteria-induced NETosis occurs in a Nox-dependent manner.

**Figure 8:**
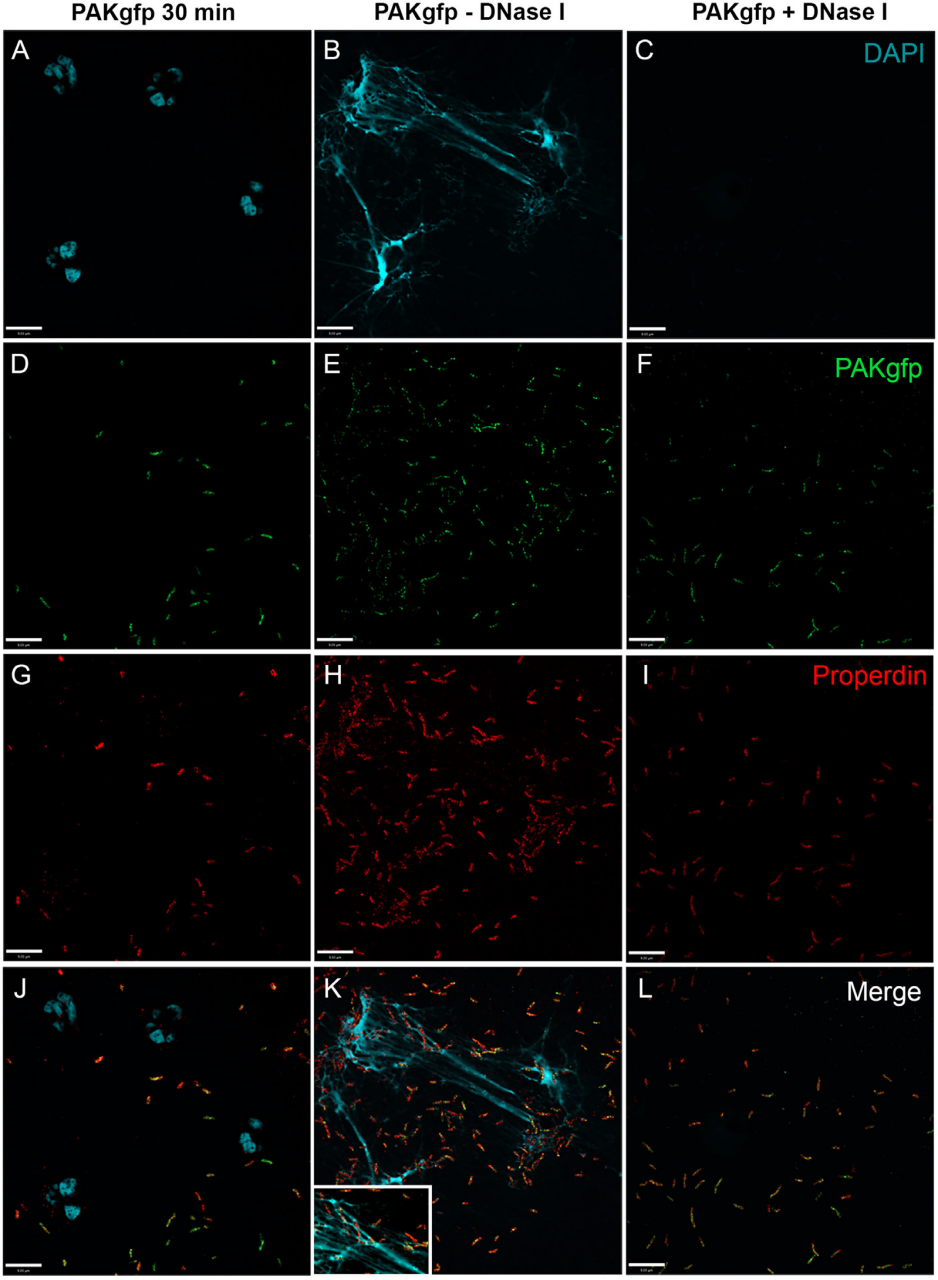
**CFP binds bacteria and NETs during *P. aeruginosa-*induced NETosis**. Neutrophils were activated in the absence of serum with PAKgfp for 30 min **(A,D,G,J)**, 240 min **(B,E,H,K)**, and 240 min with DNase I **(C,F,I,L)**. Neutrophil CFP [**(G–I)**; red] binds to PAKgfp [**(D–F)**; green]. Merged images **(J–L)**. Representative images from one of three independent experiments are shown. Confocal images were taken with a 60×/1.35 oil immersion objective. **(K)** with 2× magnified inset of a representative area. Scale bar, 9.00 μm.

To determine CFP deposition during NETosis, specimens were immunostained. In the absence of serum, CFP was detected on both bacteria and NETs, and DNAse I treatment abolished CFP deposition (Figure 8; Figure 8K with 2× magnified inset of a representative area). These results indicate that during *P. aeruginosa*-induced NETosis CFP is released from the neutrophils and deposits on NETs and bacteria.

### *P. aeruginosa*-Induced NETosis Activates Complement on NETs

**Figure 9:**
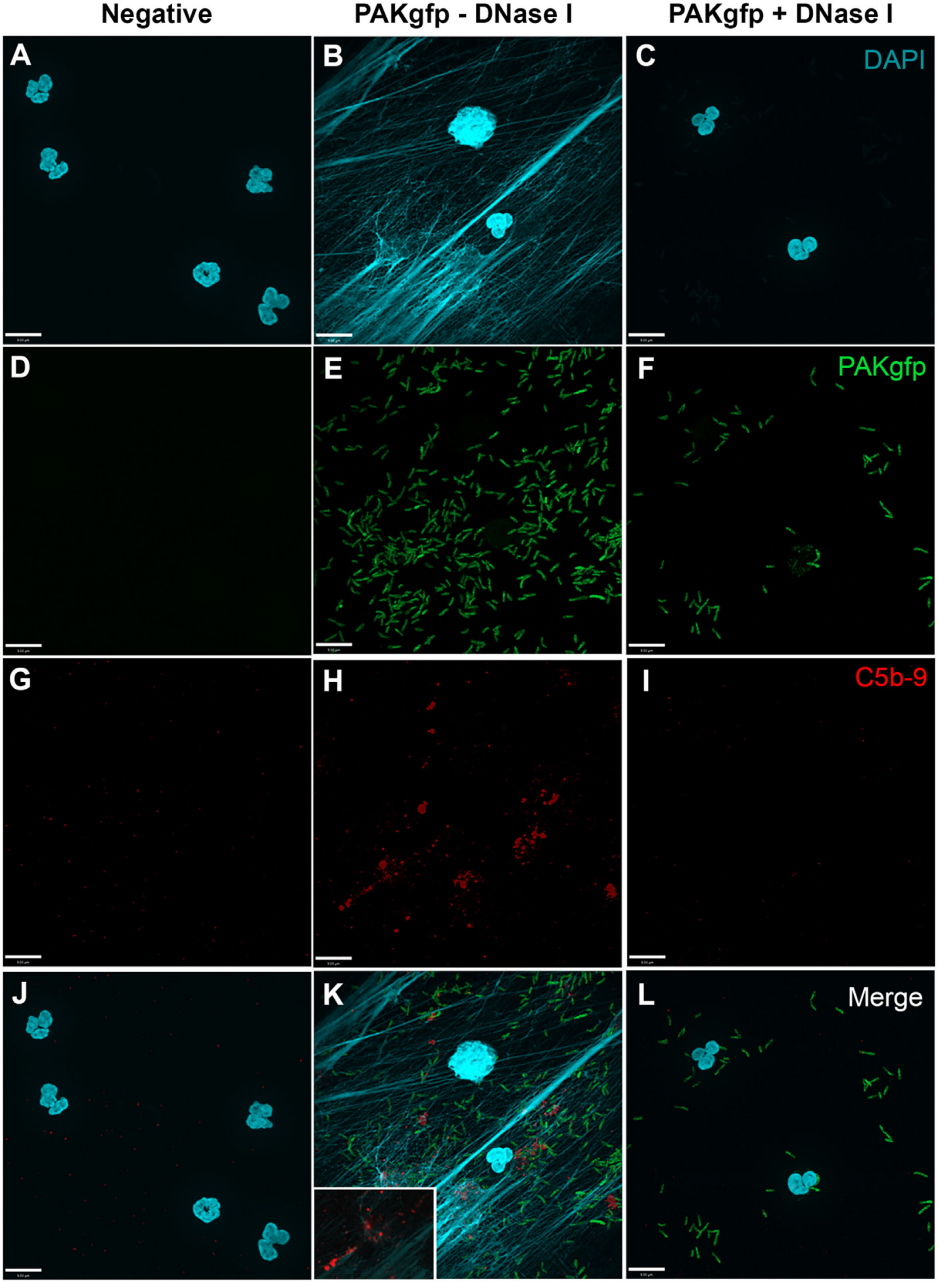
**C5b–9 deposits on *P. aeruginosa-*induced NETs**. Neutrophils untreated **(A,D,G,J)**, activated with PAKgfp **(B,E,H,K)** in the presence of DNase I **(C,F,I,L)**. Addition of 20% (v/v) autologous plasma to NETs [**(A–C)**; DAPI, blue] leads to deposition of C5b–9 [**(G–I)**; red] onto NETs [**(J–L)**; merged], but not PAKgfp [**(D–F)**; green]. Representative image from three independent experiments taken with 60×/1.35 oil immersion objective is shown. **(K)** with a 2× magnified inset of a representative area. Scale bar, 9.00 μm.

To determine whether NET induction mediated by *P. aeruginosa* results in complement activation, C5b–9 deposition was determined by immunofluorescence microscopy. Images show that C5b–9 was deposited on NETs (Figure 9; Figure 9K with a 2× magnified inset of a representative area). This effect was abolished when the NET DNA lattice was removed by DNAse treatment. Thus, complement deposits on NETs during NETosis.

### *P. aeruginosa*-Induced NETosis Also Activates Complement *via* Alternative Pathway

**Figure 10:**
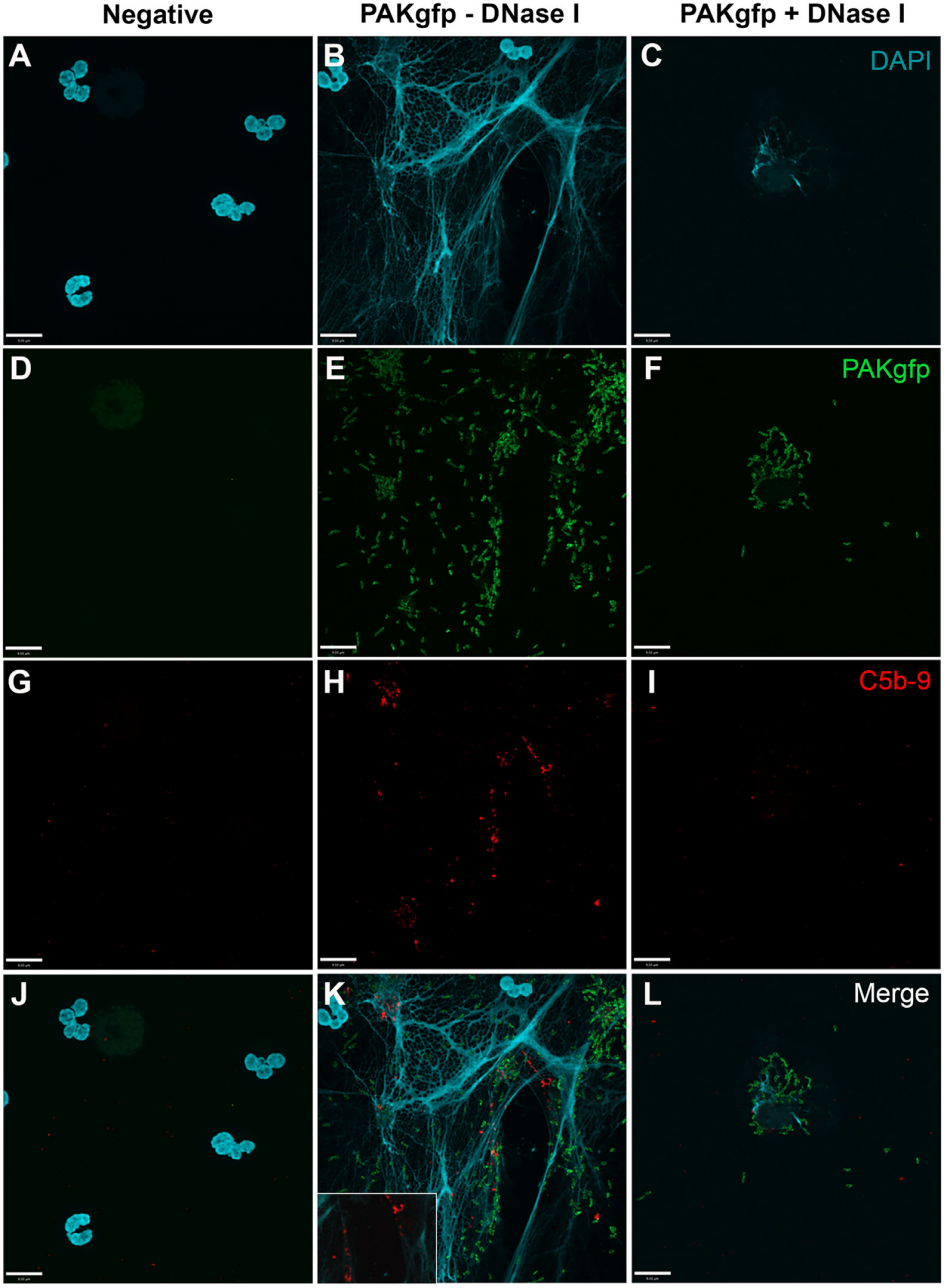
**C5b–9 deposits on *P. aeruginosa-*induced NETs *via* AP**. Neutrophils were left untreated **(A,D,G,J)**, activated with PAKgfp **(B,E,H,K)**, and treated with DNase I. A buffer exchange was performed, followed by addition of 20% (v/v) autologous plasma in AP buffer. NETs were visualized with **(A–C)** DAPI, and immunostained for [**(D–F)**; green] GFP, [**(G–I)**; red], and [**(J–L)**; merged] C5b–9. Images were taken with 60×/1.35 oil immersion objective. **(K)** with a 2× magnified inset of a representative area. Scale bar, 9.00 μm.

To determine whether the AP is involved in *P. aeruginosa-*induced NETosis and C5b–9 formation, we first examined C5b–9 deposition under AP activation conditions. Immunofluorescence microscopy analysis shows that C5b–9 is deposited on NETs (Figure 10; Figure 10K with a 2× magnified inset of a representative area).

**Figure 11:**
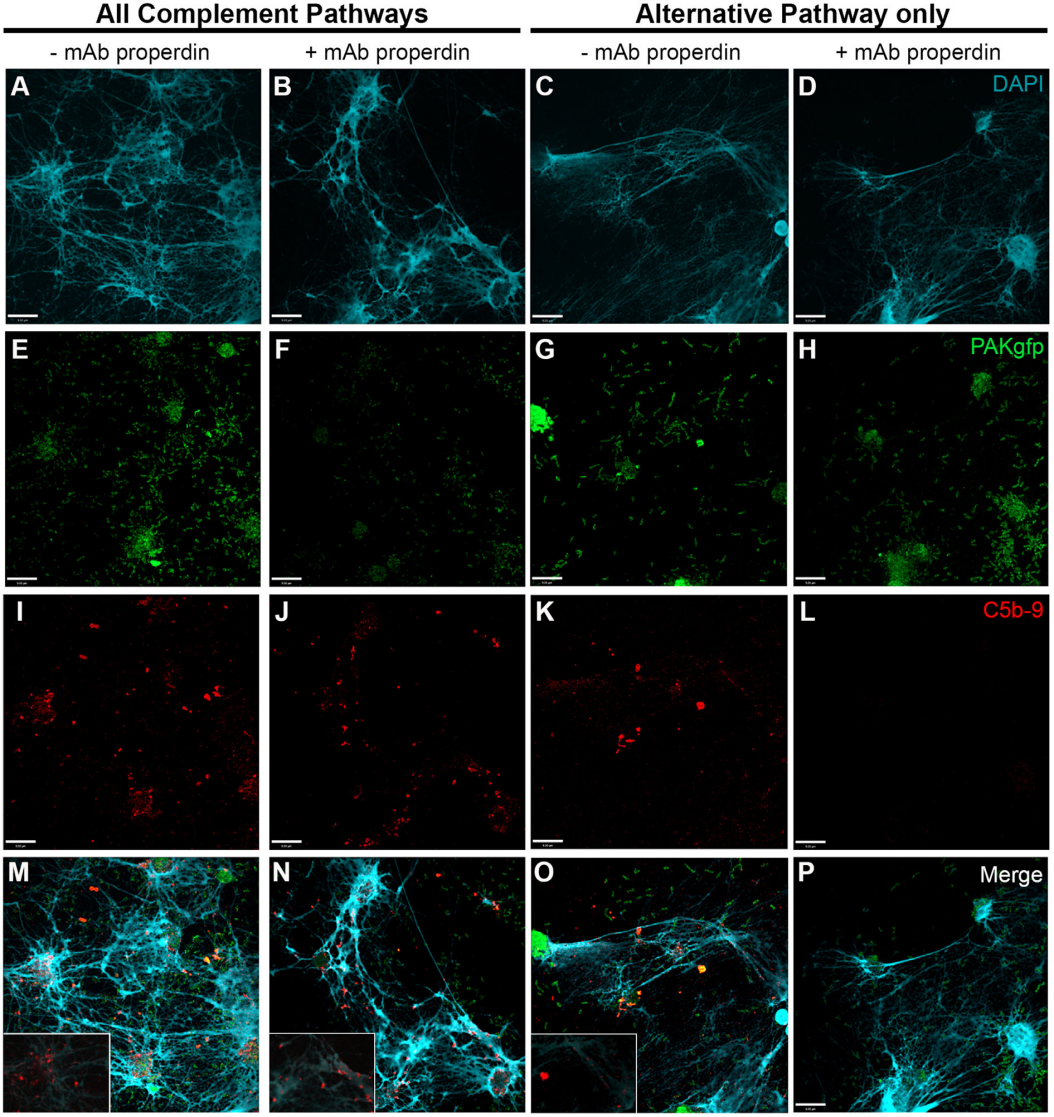
***P. aeruginosa-*induced NETosis results in complement activation on NETs *via* CFP-dependent and -independent mechanisms**. Neutrophils were activated with *P. aeruginosa* PAKgfp MOI 100 [**(E–H)**; green] to induce the formation of NETs [**(A–D)**; blue]. Samples were left in RPMI buffer (left two columns) followed by addition of 20% (v/v) autologous plasma in RPMI buffer, or in 20% plasma in AP buffer (AP activation only, right two columns). Samples were immunolabeled for C5b–9 [**(I–L)**; red]. Merged images are shown in **(M–P)**. Addition of anti-properdin antibody has no effect on C5b–9 formation in RPMI buffer **(J,N)**. Incubation of samples in 20% plasma in AP buffer (AP activation only) with a anti-properdin antibody abolished C5b–9 deposition on NETs **(L,P)**. Images were taken with 60×/1.35 oil immersion objective. **(M–O)** with a 2× magnified insets of a representative area. Scale bar, 9.00 μm.

Next, we used anti-CFP antibody to test the importance of CFP under AP activation conditions (Figure S3 in Supplementary Material). Induction of NETosis was performed using PAKgfp at an MOI of 100. After NET induction with *PAKgfp*, samples were maintained in complement competent RPMI media with the addition of 20% (v/v) autologous plasma (Figure 11 – first column; Figure 11M with a 2× magnified inset of a representative area). The addition of anti-CFP antibody to these samples did not change the formation and deposition of C5b–9 (Figure 11 – second column; Figure 11N with a 2× magnified inset of a representative area). Next, we incubated neutrophils with 20% (v/v) plasma in AP buffer. C5b–9 deposition was detected on NETs (Figure 11 – third column; Figure 11O with a 2× magnified inset of a representative area). Adding 4 μg/ml anti-CFP antibody before incubating bacteria-induced NETs with plasma inhibited C5b–9 deposition on NETs (Figure 11 – fourth column). Taken together, these data show that complement activation and progression to C5b–9 formation on NETs occurs *via* AP and non-AP pathways, and that AP activation depends on CFP.

## Discussion

Over the past decade, the ability of neutrophils to generate NETs has led to studies attempting to determine their function and involvement in disease. Although extensive studies have been performed, the exact functions of NETs, and their mechanism of action, remain to be completely elicited. NETs have been identified in several diseases that are associated with complement activation (1, 13, 14, 17, 33). In this study, we sought to understand the involvement of the complement system in the context of bacteria and NETs.

The use of varying reagents to induce NETosis has been established in many studies (4, 5). Applying these conditions, we found that PMA, but not C5a and fMLP, induces NETosis *via* the production of ROS. In order to obtain a more physiological impression of the mechanism of NETosis and the interplay with components of the complement system, we incorporated the use of *P. aeruginosa* in further studies. As previously seen (28–30), bacteria are capable of inducing Nox-dependent NETosis in a load-dependent manner.

Neutrophils recruited to sites of inflammation are a major determinant of AP activation (1). Neutrophil stimulation with PMA, C5a, and fMLP resulted in a quick release of the complement proteins CFP, C3, and CFB. These proteins are critical for the assembly of the AP convertase C3bBb, where CFP functions as a stabilizer (26). After demonstrating neutrophil release of complement proteins during NETosis, we confirmed that complement proteins became entangled within the NET structures. C5b–9 was deposited on the NETs, independent of the stimulus (PMA and *PAKgfp*). Deposition was abrogated by the use of DNAse, signifying the requirement of the NET lattices for the generation of C5b–9. This finding supports a role for NETs in inducing and/or enhancing complement activation, which is in keeping with the recently published observation that NETs can activate and deposit complement AP components (25). CFP also binds to DNA exposed by necrotic or apoptotic cells, and neutrophil-secreted CFP has been linked to a positive feedback loop between neutrophil and complement activation (1, 34–37). We also found CFP deposition on NETs, and the use of an anti-CFP antibody blocking the AP allowed us to identify the complement pathways involved in NET-mediated C5b–9 activation (38).

Complement factor P blockade was efficient in preventing terminal pathway activation when experimental conditions limited complement activation (i.e., C5b–9) on NETs to the AP. When the classical and lectin pathways were also allowed to be activated, this AP-specific blocking effect was lost. Fluorescence microscopy data suggest the possibility of CFP-mediated C5b–9 formation on NETs – data consistent with recent studies, indicating that both AP- and non-AP-mediated complement activation can occur on NETs (1, 5, 25, 37, 39, 40). CFP binding to targets *via* C3 fragments (alone or in context of the C3-/C5-convertases) is also possible.

Taken together, our results demonstrate that the “AP tool kit” present in the neutrophils are released upon stimulus, and deposits on targets, such as NETs. In the presence of plasma, NET formation results in terminal pathway activation *via* both CFP-dependent and -independent mechanisms. In NET-mediated diseases, the formation of NETs might trigger complement activation and exert secondary effects, such as cell injury and death. This is of great importance, as therapeutic complement inhibitors (e.g., eculizumab) are now available for clinical use (41, 42).

## Author Contributions

JY designed and carried out the experiments, interpreted data, and wrote the first draft of the manuscript; AC, MR, and FP contributed to designing and carrying out experiments, interpreting data, and writing the manuscript; DD provided technical assistance in designing and carrying out some of the experiments; MU provided the PAKgfp strain and contributed interpreting data; WK contributed designing experiments and interpreting data; NP and CL designed experiments, supervised the study, interpreted data, and wrote the final manuscript.

## Conflict of Interest Statement

The authors declare that the research was conducted in the absence of any commercial or financial relationships that could be construed as a potential conflict of interest.

## Supplementary Material

Figure S1 **PMA and fMLP, but not C5a, induce ROS production in neutrophils**. Neutrophils were activated with **(A)** C5a (1 or 2 μM), **(B)** fMLP (1 or 2 μM), and **(C)** PMA (20 nM) and analyzed by flow cytometry for oxidative burst using dihydrorhodamine (DHR) 123. A significant ROS production was only observed for fMLP (1 μM) and PMA (20 nM). Results are given as median fluorescence intensity (MFI) from three independent experiments. Student’s *t*-test, **p* < 0.01.

Figure S2 **Confocal images showing PMA-mediated kinetics of NETosis**. Neutrophils were activated with PMA (20 nM) to induce NET formation. Samples were fixed with 4% (w/v) paraformaldehyde and stained with DAPI for microscopy. **(A)** Four distinct nuclear morphologies (lobulated, delobulated, decondensed nuclei; NETs) can be identified during NETosis. **(B)** Percentage difference for nuclear morphologies was identified through manual counting of at least 118–220 cells from five different focal planes at 40× magnification. Data are presented as mean ± SEM from three independent experiments. Statistical significance is shown only if percentage of nuclear morphology is significantly different compared to all other morphologies at the same time point. Two-way ANOVA with Tukey’s multiple comparison test, **p* < 0.05, ^#^*p* < 0.001, ^$^*p* < 0.0001.

Figure S3 **Anti-CFP antibody concentrations of >4 μg/ml block AP-mediated complement activation**. Serial dilutions of a mouse monoclonal anti-properdin antibody were performed to determine the antibody concentration required to completely inhibit complement AP as determined by rabbit erythrocyte lysis. This was achieved using concentrations >4 μg/ml. Data are presented as mean ± SEM from three independent experiments. Student’s *t*-test, **p* < 0.05, ***p* < 0.01.

## References

[1] Kolaczkowska E Kubes P. Neutrophil recruitment and function in health and inflammation. Nat Rev Immunol (2013) 13:159–75. 10.1038/nri3399 23435331

[2] Nauseef WM Borregaard N. Neutrophils at work. Nat Immunol (2014) 15:602–11. 10.1038/ni.2921 24940954

[3] Urban CF Ermert D Schmid M Abu-Abed U Goosmann C Nacken W Neutrophil extracellular traps contain calprotectin, a cytosolic protein complex involved in host defense against *Candida albicans*. PLoS Pathog (2009) 5:e1000639. 10.1371/journal.ppat.1000639 19876394 PMC2763347

[4] Cheng OZ Palaniyar N. NET balancing: a problem in inflammatory lung diseases. Front Immunol (2013) 4:1. 10.3389/fimmu.2013.00001 23355837 PMC3553399

[5] Zawrotniak M Rapala-Kozik M. Neutrophil extracellular traps (NETs) – formation and implications. Acta Biochim Pol (2013) 60:277–84. 23819131

[6] Fuchs TA Abed U Goosmann C Hurwitz R Schulze I Wahn V Novel cell death program leads to neutrophil extracellular traps. J Cell Biol (2007) 176:231–41. 10.1083/jcb.200606027 17210947 PMC2063942

[7] Douda DN Yip L Khan MA Grasemann H Palaniyar N Akt is essential to induce NADPH-dependent NETosis and to switch the neutrophil death to apoptosis. Blood (2014) 123:597–600. 10.1182/blood-2013-09-526707 24458280

[8] Douda DN Grasemann H Pace-Asciak C Palaniyar N A lipid mediator hepoxilin A3 is a natural inducer of neutrophil extracellular traps in human neutrophils. Mediators Inflamm (2015) 2015:520871 10.1155/2015/520871 25784781 PMC4345265

[9] Douda DN Khan MA Grasemann H Palaniyar N SK3 channel and mitochondrial ROS mediate NADPH oxidase-independent NETosis induced by calcium influx. Proc Natl Acad Sci U S A (2015) 112:2817–22. 10.1073/pnas.1414055112 25730848 PMC4352781

[10] Yousefi S Mihalache C Kozlowski E Schmid I Simon HU. Viable neutrophils release mitochondrial DNA to form neutrophil extracellular traps. Cell Death Differ (2009) 16:1438–44. 10.1038/cdd.2009.96 19609275

[11] Papayannopoulos V Zychlinsky A. NETs: a new strategy for using old weapons. Trends Immunol (2009) 30:513–21. 10.1016/j.it.2009.07.011 19699684

[12] Clark SR Ma AC Tavener SA McDonald B Goodarzi Z Kelly MM Platelet TLR4 activates neutrophil extracellular traps to ensnare bacteria in septic blood. Nat Med (2007) 13:463–9. 10.1038/nm1565 17384648

[13] Kessenbrock K Krumbholz M Schonermarck U Back W Gross WL Werb Z Netting neutrophils in autoimmune small-vessel vasculitis. Nat Med (2009) 15:623–5. 10.1038/nm.1959 19448636 PMC2760083

[14] Hakkim A Furnrohr BG Amann K Laube B Abed UA Brinkmann V Impairment of neutrophil extracellular trap degradation is associated with lupus nephritis. Proc Natl Acad Sci U S A (2010) 107:9813–8. 10.1073/pnas.0909927107 20439745 PMC2906830

[15] Douda DN Jackson R Grasemann H Palaniyar N. Innate immune collectin surfactant protein D simultaneously binds both neutrophil extracellular traps and carbohydrate ligands and promotes bacterial trapping. J Immunol (2011) 187:1856–65. 10.4049/jimmunol.1004201 21724991

[16] Villanueva E Yalavarthi S Berthier CC Hodgin JB Khandpur R Lin AM Netting neutrophils induce endothelial damage, infiltrate tissues, and expose immunostimulatory molecules in systemic lupus erythematosus. J Immunol (2011) 187:538–52. 10.4049/jimmunol.1100450 21613614 PMC3119769

[17] Kaplan MJ Radic M. Neutrophil extracellular traps: double-edged swords of innate immunity. J Immunol (2012) 189:2689–95. 10.4049/jimmunol.1201719 22956760 PMC3439169

[18] Bellac CL Dufour A Krisinger MJ Loonchanta A Starr AE Auf Dem Keller U Macrophage matrix metalloproteinase-12 dampens inflammation and neutrophil influx in arthritis. Cell Rep (2014) 9:618–32. 10.1016/j.celrep.2014.09.006 25310974

[19] Jin L Batra S Douda DN Palaniyar N Jeyaseelan S. CXCL1 contributes to host defense in polymicrobial sepsis via modulating T cell and neutrophil functions. J Immunol (2014) 193:3549–58. 10.4049/jimmunol.1401138 25172493 PMC4170008

[20] Muller S Radic M Citrullinated autoantigens: from diagnostic markers to pathogenetic mechanisms. Clin Rev Allergy Immunol (2014) 49:232–9. 10.1007/s12016-014-8459-2 25355199

[21] Riedl M Fakhouri F Le Quintrec M Noone DG Jungraithmayr TC Fremeaux-Bacchi V Spectrum of complement-mediated thrombotic microangiopathies: pathogenetic insights identifying novel treatment approaches. Semin Thromb Hemost (2014) 40:444–64. 10.1055/s-0034-1376153 24911558

[22] Grayson PC Kaplan MJ At the bench: neutrophil extracellular traps (NETs) highlight novel aspects of innate immune system involvement in autoimmune diseases. J Leukoc Biol (2015) 99(2):253–64. 10.1189/jlb.5BT0615-247R 26432901 PMC4718195

[23] Palaniyar N Mall MA Taube C Worgall S Grasemann H New developments in cystic fibrosis airway inflammation. Mediators Inflamm (2015) 2015:769425 10.1155/2015/769425 26221065 PMC4499624

[24] Yildiz C Palaniyar N Otulakowski G Khan MA Post M Kuebler WM Mechanical ventilation induces neutrophil extracellular trap formation. Anesthesiology (2015) 122:864–75. 10.1097/ALN.0000000000000605 25665049

[25] Wang H Wang C Zhao MH Chen M. Neutrophil extracellular traps can activate alternative complement pathways. Clin Exp Immunol (2015) 181:518–27. 10.1111/cei.12654 25963026 PMC4557387

[26] Noris M Remuzzi G. Overview of complement activation and regulation. Semin Nephrol (2013) 33:479–92. 10.1016/j.semnephrol.2013.08.001 24161035 PMC3820029

[27] Cortes C Ohtola JA Saggu G Ferreira VP Local release of properdin in the cellular microenvironment: role in pattern recognition and amplification of the alternative pathway of complement. Front Immunol (2012) 3:412 10.3389/fimmu.2012.00412 23335922 PMC3547370

[28] Young RL Malcolm KC Kret JE Caceres SM Poch KR Nichols DP Neutrophil extracellular trap (NET)-mediated killing of *Pseudomonas aeruginosa*: evidence of acquired resistance within the CF airway, independent of CFTR. PLoS One (2011) 6:e23637. 10.1371/journal.pone.0023637 21909403 PMC3164657

[29] Khatua B Bhattacharya K Mandal C. Sialoglycoproteins adsorbed by *Pseudomonas aeruginosa* facilitate their survival by impeding neutrophil extracellular trap through siglec-9. J Leukoc Biol (2012) 91:641–55. 10.1189/jlb.0511260 22241833

[30] Shan Q Dwyer M Rahman S Gadjeva M. Distinct susceptibilities of corneal *Pseudomonas aeruginosa* clinical isolates to neutrophil extracellular trap-mediated immunity. Infect Immun (2014) 82:4135–43. 10.1128/IAI.02169-14 25047845 PMC4187885

[31] Cote O Clark ME Viel L Labbe G Seah SY Khan MA Secretoglobin 1A1 and 1A1A differentially regulate neutrophil reactive oxygen species production, phagocytosis and extracellular trap formation. PLoS One (2014) 9:e96217. 10.1371/journal.pone.0096217 24777050 PMC4002474

[32] Heinen S Pluthero FG Van Eimeren VF Quaggin SE Licht C. Monitoring and modeling treatment of atypical hemolytic uremic syndrome. Mol Immunol (2013) 54:84–8. 10.1016/j.molimm.2012.10.044 23220071

[33] Fuchs TA Kremer Hovinga JA Schatzberg D Wagner DD Lammle B. Circulating DNA and myeloperoxidase indicate disease activity in patients with thrombotic microangiopathies. Blood (2012) 120:1157–64. 10.1182/blood-2012-02-412197 22611154 PMC3418712

[34] Schwaeble WJ Reid KB Does properdin crosslink the cellular and the humoral immune response? Immunol Today (1999) 20:17–21. 10.1016/S0167-5699(98)01376-0 10081225

[35] Kemper C Hourcade DE. Properdin: new roles in pattern recognition and target clearance. Mol Immunol (2008) 45:4048–56. 10.1016/j.molimm.2008.06.034 18692243 PMC2628304

[36] Camous L Roumenina L Bigot S Brachemi S Fremeaux-Bacchi V Lesavre P Complement alternative pathway acts as a positive feedback amplification of neutrophil activation. Blood (2010) 117:1340–9. 10.1182/blood-2010-05-283564 21063021

[37] O’Flynn J Dixon KO Faber Krol MC Daha MR Van Kooten C Myeloperoxidase directs properdin-mediated complement activation. J Innate Immun (2013) 6:417–25. 10.1159/000356980 24355864 PMC6741500

[38] Gupta-Bansal R Parent JB Brunden KR. Inhibition of complement alternative pathway function with anti-properdin monoclonal antibodies. Mol Immunol (2000) 37:191–201. 10.1016/S0161-5890(00)00047-X 10930626

[39] Leffler J Martin M Gullstrand B Tyden H Lood C Truedsson L Neutrophil extracellular traps that are not degraded in systemic lupus erythematosus activate complement exacerbating the disease. J Immunol (2012) 188:3522–31. 10.4049/jimmunol.1102404 22345666

[40] Farrera C Fadeel B. Macrophage clearance of neutrophil extracellular traps is a silent process. J Immunol (2013) 191:2647–56. 10.4049/jimmunol.1300436 23904163

[41] Rother RP Rollins SA Mojcik CF Brodsky RA Bell L. Discovery and development of the complement inhibitor eculizumab for the treatment of paroxysmal nocturnal hemoglobinuria. Nat Biotechnol (2007) 25:1256–64. 10.1038/nbt1207-1488c 17989688

[42] Legendre CM Licht C Muus P Greenbaum LA Babu S Bedrosian C Terminal complement inhibitor eculizumab in atypical hemolytic-uremic syndrome. N Engl J Med (2013) 368:2169–81. 10.1056/NEJMoa1208981 23738544

