# Supplementary figures and images for: NETosing Neutrophils Activate Complement Both on Their Own NETs and Bacteria via Alternative and Non-alternative Pathways
Source: Front Immunol. 2016 Apr 14;7:137. doi: 10.3389/fimmu.2016.00137 (PMC4831636; doi:10.3389/fimmu.2016.00137)

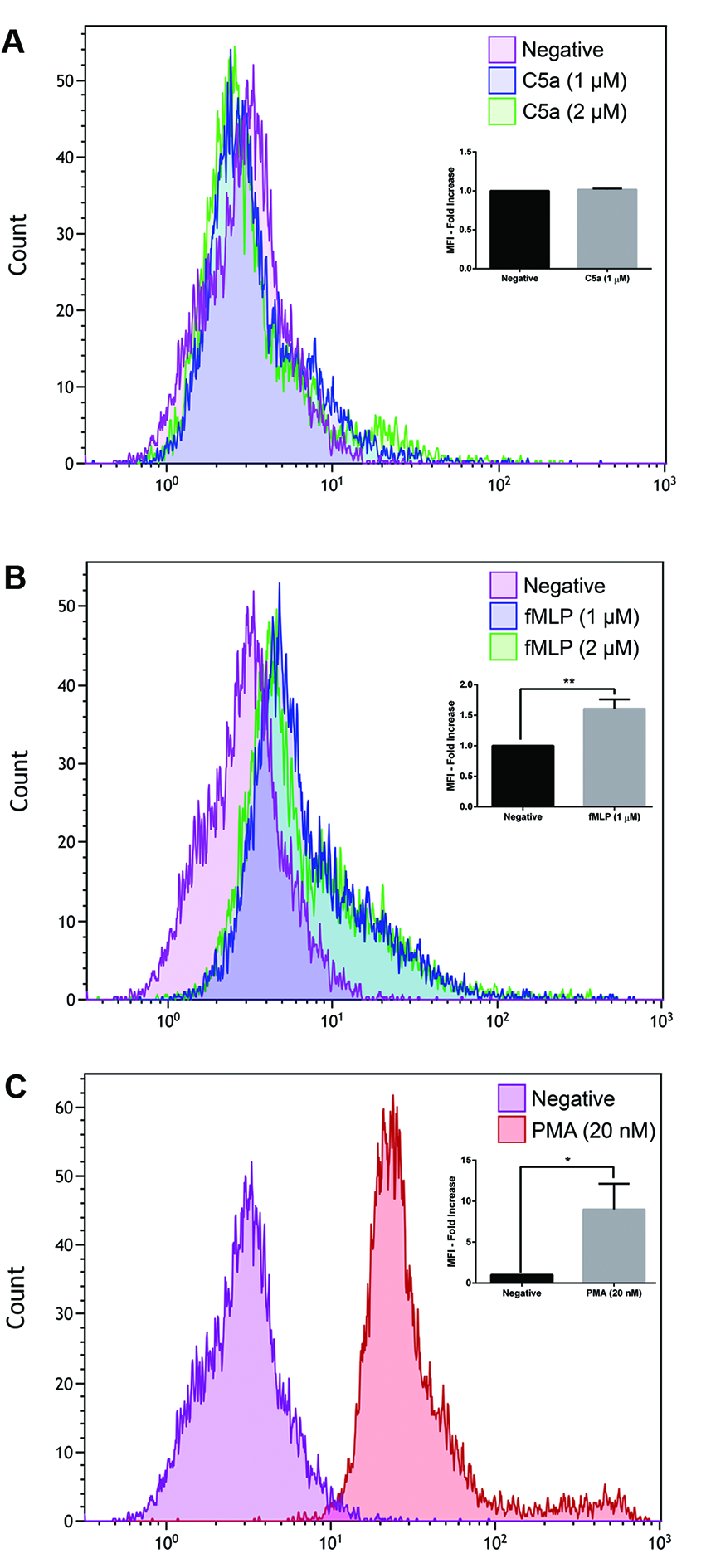

Supplement: Figure S1 — PMA and fMLP, but not C5a, induce ROS production in neutrophils. Neutrophils were activated with (A) C5a (1 or 2 μM), (B) fMLP (1 or 2 μM), and (C) PMA (20 nM) and analyzed by flow cytometry for oxidative burst using dihydrorhodamine (DHR) 123. A significant ROS production was only observed for fMLP (1 μM) and PMA (20 nM). Results are given as median fluorescence intensity (MFI) from three independent experiments. Student’s t-test, *p < 0.01. [file image_1.tif]

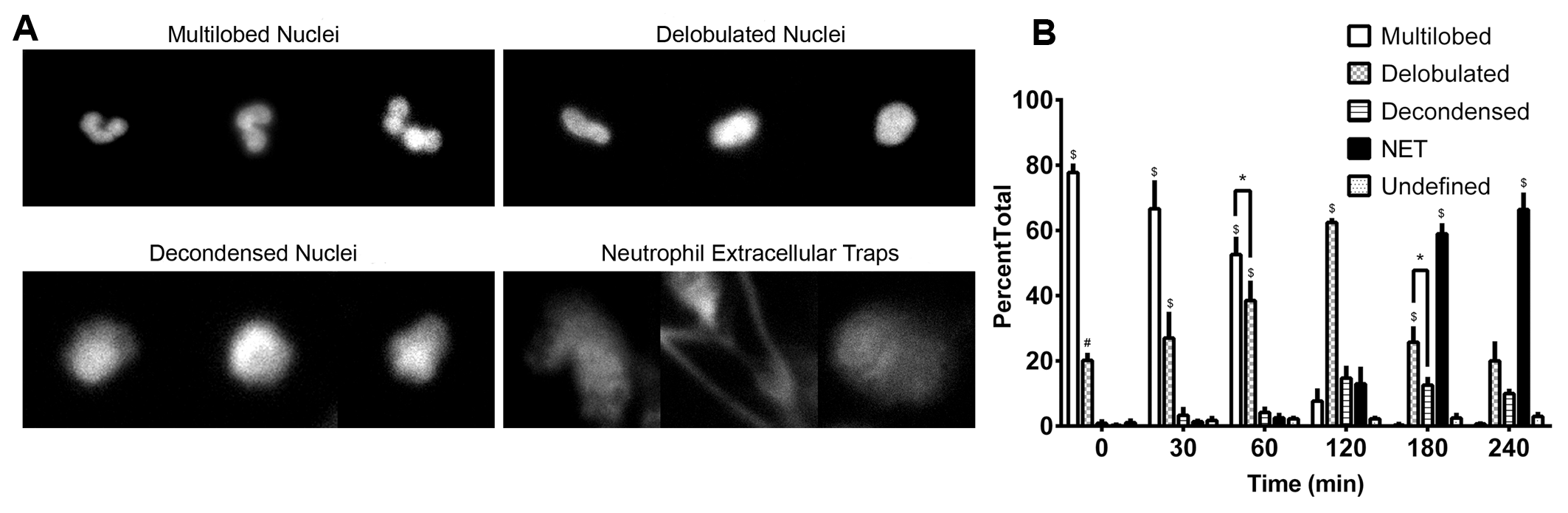

Supplement: Figure S2 — Confocal images showing PMA-mediated kinetics of NETosis. Neutrophils were activated with PMA (20 nM) to induce NET formation. Samples were fixed with 4% (w/v) paraformaldehyde and stained with DAPI for microscopy. (A) Four distinct nuclear morphologies (lobulated, delobulated, decondensed nuclei; NETs) can be identified during NETosis. (B) Percentage difference for nuclear morphologies was identified through manual counting of at least 118–220 cells from five different focal planes at 40× magnification. Data are presented as mean ± SEM from three independent experiments. Statistical significance is shown only if percentage of nuclear morphology is significantly different compared to all other morphologies at the same time point. Two-way ANOVA with Tukey’s multiple comparison test, *p < 0.05, #p < 0.001, $p < 0.0001. [file image_2.tif]

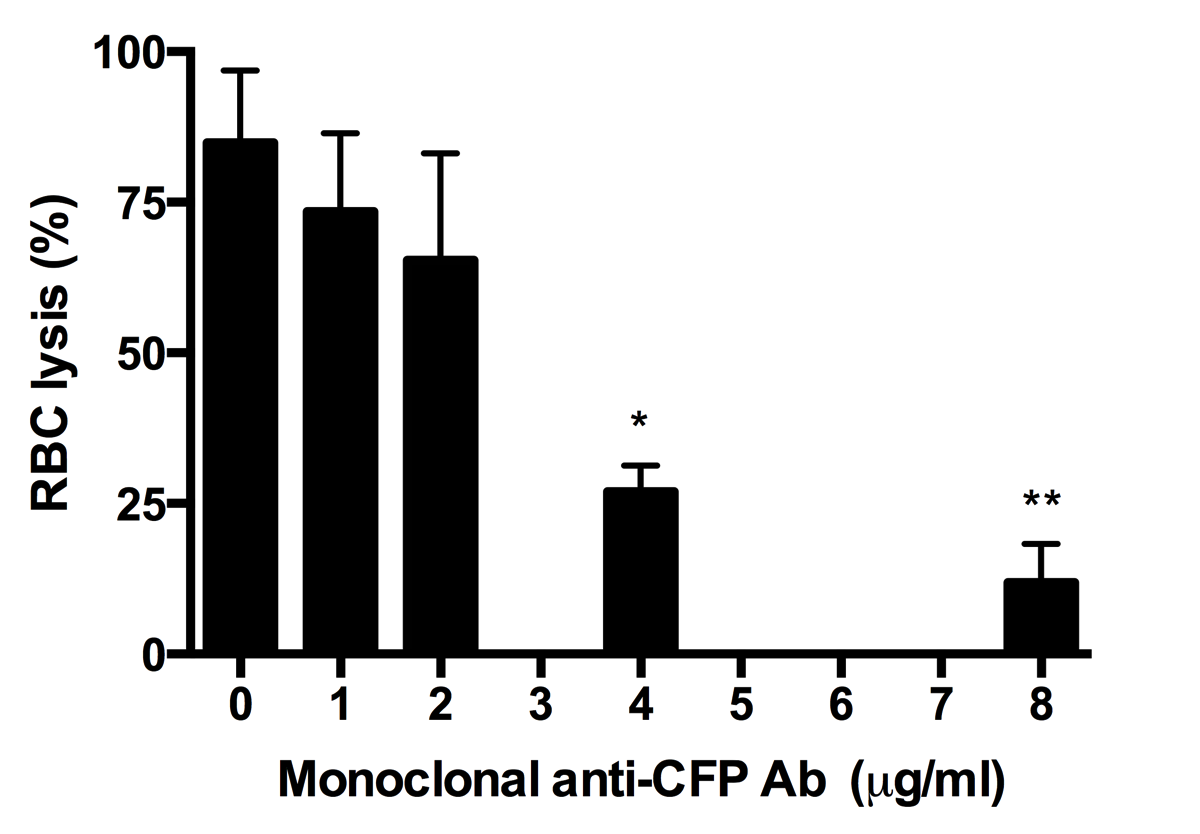

Supplement: Figure S3 — Anti-CFP antibody concentrations of >4 μg/ml block AP-mediated complement activation. Serial dilutions of a mouse monoclonal anti-properdin antibody were performed to determine the antibody concentration required to completely inhibit complement AP as determined by rabbit erythrocyte lysis. This was achieved using concentrations >4 μg/ml. Data are presented as mean ± SEM from three independent experiments. Student’s t-test, *p < 0.05, **p < 0.01. [file image_3.tiff]
